# Human Umbilical Cord Mesenchymal Stem Cells: A New Therapeutic Option for Tooth Regeneration

**DOI:** 10.1155/2015/549432

**Published:** 2015-06-02

**Authors:** Yuanwei Chen, Yongchun Yu, Lin Chen, Lanfeng Ye, Junhui Cui, Quan Sun, Kaide Li, Zhiyong Li, Lei Liu

**Affiliations:** ^1^State Key Laboratory of Oral Diseases, West China Hospital of Stomatology, Sichuan University, Chengdu 610041, China; ^2^Department of Stomatology, The First Affiliated Hospital of Guangzhou Medical College, Guangzhou 510120, China; ^3^Department of Oral & Maxillofacial Surgery, The First Affiliated Hospital, College of Medicine, Zhejiang University, Hangzhou 310003, China

## Abstract

Tooth regeneration is considered to be an optimistic approach to replace current treatments for tooth loss. It is important to determine the most suitable seed cells for tooth regeneration. Recently, human umbilical cord mesenchymal stem cells (hUCMSCs) have been regarded as a promising candidate for tissue regeneration. However, it has not been reported whether hUCMSCs can be employed in tooth regeneration. Here, we report that hUCMSCs can be induced into odontoblast-like cells *in vitro* and *in vivo*. Induced hUCMSCs expressed dentin-related proteins including dentin sialoprotein (DSP) and dentin matrix protein-1 (DMP-1), and their gene expression levels were similar to those in native pulp tissue cells. Moreover, DSP- and DMP-1-positive calcifications were observed after implantation of hUCMSCs *in vivo*. These findings reveal that hUCMSCs have an odontogenic differentiation potency to differentiate to odontoblast-like cells with characteristic deposition of dentin-like matrix *in vivo*. This study clearly demonstrates hUCMSCs as an alternative therapeutic cell source for tooth regeneration.

## 1. Introduction

Tooth loss caused by caries, periodontitis, and mechanical trauma is a major public health problem worldwide [1]. People with tooth loss have poor oral health-related quality of life involving the problems with eating, chewing, smiling, and communication [2]. Current treatments for tooth loss rely on artificial dentures, such as fixed bridges, removable dentures, and dental implants. However, compared with natural teeth, artificial dentures are nonbiological and have some disadvantages including a foreign body sensation and finite usability, all of which dissatisfy patients [3, 4]. Thus, biological teeth are considered to be necessary [5].

In the fields of stem cells and tissue regeneration, biological tooth crowns and roots have already been generated in animal studies [6, 7]. Moreover, tooth regeneration has been thought as a possible approach in the next generation of dental treatments [8]. However, one of key factors to achieve the goal is elucidation of the most suitable seed cells for tooth regeneration. Embryonic stem cells (ESCs) and adult stem cells are the two main types of stem cells for tooth regeneration [9]. Despite high proliferation and differentiation capabilities, ESCs are rarely applied in clinical practice because of possible tumorigenesis and ethical issues [10]. Thus, recent studies on seed cells for tooth regeneration have mainly focused on adult stem cells. Among the adult stem cells, dental stem cells have been considered as a candidate for tooth regeneration. These include the dental pulp stem cells, stem cells from exfoliated deciduous teeth, periodontal ligament stem cells, stem cells from apical papilla, and dental follicle progenitor cells [11]. All of these cells have already been proved owning multipotent and odontogenic differentiation potentials, and some of them have successfully applied into tooth regeneration studies [12]. However, the use of dental stem cells has several potential limitations. The primary challenge is the limited availability of dental stem cells, especially from those who are agomphious. In addition, cellular rejection and ethical issues in allogeneic therapy further hinder the clinical application of dental stem cells [13, 14]. On the other hand, induction of nondental ectomesenchyme odontogenesis by coculture with oral epithelium has provided the experimental basis to use nondental adult stem cells for tooth regeneration [15]. Li et al. reported bone marrow mesenchymal stem cells (BMMSCs) produce tooth-like structures after coculture with oral epithelial cells derived from rat embryos [16], suggesting the possibility of tooth regeneration using BMMSCs. However, invasive and painful procedures to harvest BMMSCs are difficult for people including those who require dental treatment. Therefore, a more practical and suitable kind of seed cells is needed for tooth regeneration.

Since Romanov et al. isolated the mesenchymal stem cells (MSCs) from the human umbilical cord [17], these stem cells have gained significant attention, and many advantages of hUCMSCs have been recognized. Firstly, hUCMSCs are multipotent with high capabilities for differentiation and proliferation [18]. Secondly, there is no limitation of cell source for hUCMSCs, which is a major hurdle for other stem cell types. In fact, human cord blood banks have been established worldwide, which provide a reliable source of hUCMSCs [19, 20]. More importantly, human umbilical cords would otherwise be discarded after childbirth, and there are no invasive and/or painful procedures for both the mother and infant during collection, so there are fewer ethical issues [21]. In addition, because of the protection of the placental barrier, there is a lower risk of viral contamination compared with other sources of adult stem cells [22].

hUCMSCs can differentiate into cardiomyocytes, skeletal muscle cells, endotheliocytes, and neurons and have been applied in studies of osteochondral, musculoskeletal, and bone tissue regeneration [23–26]. However, it has not been reported whether hUCMSCs can be applied in tooth regeneration.

Therefore, in this study, we examined whether hUCMSCs have an odontogenic differentiation potential. We used Sprague-Dawley (SD) rat tooth germ cell conditioned medium (TGC-CM) and human tooth dentin matrix (hTDM) to induce the hUCMSCs into odontoblast-like cells* in vitro* and determined whether the hUCMSCs can be differentiated into odontoblast-like cells* in vivo*.

## 2. Materials and Methods

This study followed the guidelines in the Declaration of Helsinki and the International Guiding Principles for Animal Research and Law for Management of Experimental Animal. The Research Ethics Board for both human samples and animal experiments established by the Ethics Committee of the West China Hospital of Stomatology, Sichuan University, examined the proposed research protocol for this project and found it to be ethically acceptable.

### 2.1. hUCMSCs Isolation, Culture, and Identification

Fresh human umbilical cords were collected from full-term births by cesarean section from Chengdu Women's and Children's Central Hospital and stored in phosphate buffered saline (PBS) containing 100 U/mL penicillin and 100 U/mL streptomycin, which was informed consent of the parents and conducted following approval of Sichuan University Ethics Committee. The human umbilical cords were processed within 4 hours and were assigned to testing groups and control groups at random. We applied collagenase/trypsin method (0.2% collagenase and 0.25% trypsin, both purchased from Sigma, USA) and explant culture to isolated hUCMSCs from Wharton's jelly [27]. The cells were cultured by LG-DMEM/F12 (Invitrogen, USA) containing 10% fetal bovine serum (FBS, Gibco, USA), 100 U/mL penicillin (Hyclone, USA), and 100 U/mL streptomycin (Hyclone, USA), and the medium was changed every 2-3 days. Following 2-3 passages, immunohistochemistry was performed as per published protocols [28]. For adipogenic differentiation, passage three (P3) hUCMSCs were seeded at a density of 1 × 10^4^ cells/well in 6-well plates and washed with PBS twice when the cells reached 80% confluence. The cells were maintained in LG-DMEM/F12 containing 10% FBS, 1 *μ*M dexamethasone (Sigma, USA), 5 *μ*g insulin (Sigma, USA), 0.5 mM 3-isobutyl-1-methylxanthine (Sigma, USA), and 0.2 mM indomethacin (Sigma, USA). Half medium change was performed every 2-3 days. The cells were induced for 2 weeks, stained with Oil red O (Sigma, USA), and then observed under a microscope (CKX41, Olympus, Japan). For osteogenic differentiation, P3 hUCMSCs were seeded at a density of 1 × 10^4^ cells/well in 6-well plates and cultured in LG-DMEM/F12 containing 10% FBS for 2 days and washed with PBS twice. Then, the cells were maintained in the osteogenic induction medium consisting of LG-DMEM/F12 containing 10% FBS, 10 mM *β*-glycerophosphate (Sigma, USA), 10^−8^ mol/L dexamethasone, and 50 *μ*g/mL ascorbic acid (Sigma, USA). The medium was changed every 2-3 days for 3 weeks until a black opaque area was observed under a microscope and white nodules were observed by the naked eye. Alizarin red S staining was applied to detect the calcium nodules.

### 2.2. Differentiation of hUCMSCs into Odontoblast-Like Cells with TGC-CM* In Vitro*

The isolation of tooth germs of SD rats and preparation of TGC-CM were performed as described previously [28, 29]. Briefly, The mandibular first molar germs were dissected from postnatal 0.5 neonatal SD rats, diced into cubes of about 1 mm^3^, digested with 0.2% collagenase for 40–60 min at 37°C, and neutralized with *α*-MEM (Hyclone, USA) containing 10% FBS, 100 U/mL penicillin, and 100 U/mL streptomycin. The digested cells were cultured by culture medium *α*-MEM containing 10% FBS, 100 U/mL penicillin, and 100 U/mL streptomycin. The medium was changed 2-3 days. The substituted conditioned medium was collected and centrifuged at 1000 rpm for 5 minutes. The supernatant was passed through a 0.22 *μ*m bacterial filter and mixed with LG-DMEM/F12 (Invitrogen, USA) containing 10% FBS, 100 U/mL penicillin, and 100 U/mL streptomycin at 1 : 1 ratio to obtain the TGC-CM.

Passage 2 (P2) hUCMSCs were seeded at a density of 1 × 10^4^ cells/well in 6-well dishes and cultured for 24 hours. After washing the cells with PBS three times, the TGC-CM was added to induce the hUCMSCs into odontoblast-like cells. The TGC-CM was changed every other day and the morphological changes of the cells were photographed under a microscope. Control hUCMSCs were cultured in LG-DMEM/F12 containing 10% FBS, 100 U/mL penicillin, and 100 U/mL streptomycin.

### 2.3. Preparation of Human Tooth Dentin Matrix (hTDM)

TDM was obtained from healthy single root premolars extracted for orthodontic reasons with informed consent of the patients. And the procedures to treat the teeth were processed by previous study [30]. Briefly, the roots of donated teeth were cut half and stored in sterile deionized water for 5-6 hours and then were oscillated by ultrasonicator at 80 Hz for 5-6 minutes before changing the sterile deionized water every hour. Then the roots were treated in 17%, 10% and 5% EDTA each for 6 minutes to remove the smear layer and rinsed with sterile deionized water for 5 min and then immersed in PBS containing 100 U/mL penicillin and 100 U/mL streptomycin for 72 hours following rinsing with sterile deionized water for 5 min and stored in LG-DMEM/F12 culture medium containing 100 U/mL penicillin and 100 U/mL streptomycin. After this, hematoxylin and eosin (HE) staining was performed to check whether the fiber tracts in hTDM became loose and the smear layer had been removed. And Masson's trichrome staining was performed to detect whether collagen fibers still existed in hTDM. And for MTT assay, the P2 hUCMSCs were seeded at a density of 5 × 10^3^ cells/well in 24-well dishes which contained hTDM, and the cells were cultured for 1–8 days. MTT solution (40 *μ*L) was added to each well, and the cells were incubated at 37°C in a humidified atmosphere with 5% CO_2_ for 3.5 h. Then, the medium was aspirated, and 200 *μ*L of dimethyl sulfoxide was added to dissolve the blue crystals that formed in the cells. After gentle agitation for 10 min, 100 *μ*L of the solution in each well was transferred to a 96-well plate. The optical density was determined with a multiplate reader at a wavelength of 570 nm. Control hUCMSCs were cultured without hTDM.

### 2.4. Differentiation of hUCMSCs under the Odontogenic Microenvironment Provided by the hTDM* In Vitro* and* Vivo*

For* in vitro* study, hUCMSCs were seeded at a density of 5 × 10^4^ cells/well in 6-well plates containing hTDM, and negative control cells were seeded at a same density in 6-well plates without hTDM. Both of testing and control groups were cultured in culture medium LG-DMEM/F12 containing 10% FBS, 100 U/mL penicillin, and 100 U/mL streptomycin. The culture medium was changed each other day and the cells were harvested after 14 days for analyses. For* in vivo* study, the hUCMSCs were seeded at a density of 5 × 10^4^ cells/well in 6-well plates containing hTDM and cultured in LG-DMEM/F12 for 24 hours at first. Then twenty hTDM-hUCMSC composites were implanted subcutaneously into the backs of nude mice under anesthesia. After 8 weeks, the implants were extracted and subjected to visual observation. HE staining, Masson's trichrome staining, and immunohistochemistry were performed to evaluate odontogenic differentiation of hUCMSCs* in vivo*.

### 2.5. Immunocytochemistry

For immunocytochemical analysis, the cells were fixed with 4% paraformaldehyde for 15 minutes. Immunocytochemistry was performed with streptavidin-biotin complex method according to the manufacturer's protocol. Antibodies against CD105 (1 : 100), CD29 (1 : 100), CD44 (1 : 100), CD34 (1 : 100), CD45 (1 : 100), CD31 (1 : 100), DSP (1 : 200), and DMP1 (1 : 100) were used in this study. The antibodies against DSP and DMP1 were purchased from Santa Cruz (USA). Other antibodies were purchased from ZSGB-BIO (China). Samples were photographed under an Olympus CKX41 microscope.

### 2.6. Western Blotting

hUCMSCs were collected and washed with PBS. Then, the cells were lysed with lysis buffer for 30 minutes. The proteins were separated by 10% SDS-polyacrylamide gel electrophoresis and then transferred to cellulose membranes. The membranes were incubated with gentle agitation at 37°C with primary antibodies *β*-actin (1 : 1000, Santa Cruz, USA), DMP-1 (1 : 100, Santa Cruz, USA), and DSP (1 : 200, Santa Cruz, USA). Then the membranes were incubated with gentle agitation for 2 hours at 37°C with horseradish peroxidase-conjugated secondary antibody diluted in 5% skim milk powder at 1 : 7500. After washing in Tris-Buffered Saline Tween-20 (Beyotime, China) for three times (10 minutes each wash), the membranes were developed by an ECL western blotting detection system. Immunoreactive proteins were then detected by ChemiDoc MP System #170-8280 (Bio-Rad, USA). Images were captured and analyzed by Quantity One software (Bio-Rad, USA).

### 2.7. Quantitative PCR

Total RNA was extracted with RNAiso Reagent (TaKaRa, Japan). The RNA was reversed-transcribed to cDNA using PrimeScript RT reagent Kit Perfect Real Time (TaKaRa, Japan). Quantitative PCR was performed in ABI PRISM 7300 Sequence Detection System (Applied Biosystems, USA). The relative expression levels for the target gene were evaluated using the 2^−ΔΔCT^ method [31]. *β*-actin gene expression was used for normalization of each sample. The primer pairs used for RT-qPCR were showed in Table 1.

### 2.8. Scanning Electron Microscope (SEM)

hTDM was observed by scanning electron microscope (SEM) (Inspect F, FEI, Netherlands). Briefly, hTDM was washed with PBS for three times, and then it was fixed with 2.5% glutaraldehyde at 0°C and dehydrated and dried in a critical-point dryer. Finally, it was observed and photographed by SEM.

### 2.9. Statistical Analysis

All quantitative data are expressed as the mean ± SD. Statistical analyses were performed using one-way analysis of variance using SPSS software. A value of *P* < 0.05 was considered to be statistically significant.

## 3. Results

### 3.1. Isolation, Culture, and Identification of hUCMSCs

**Figure 1:**
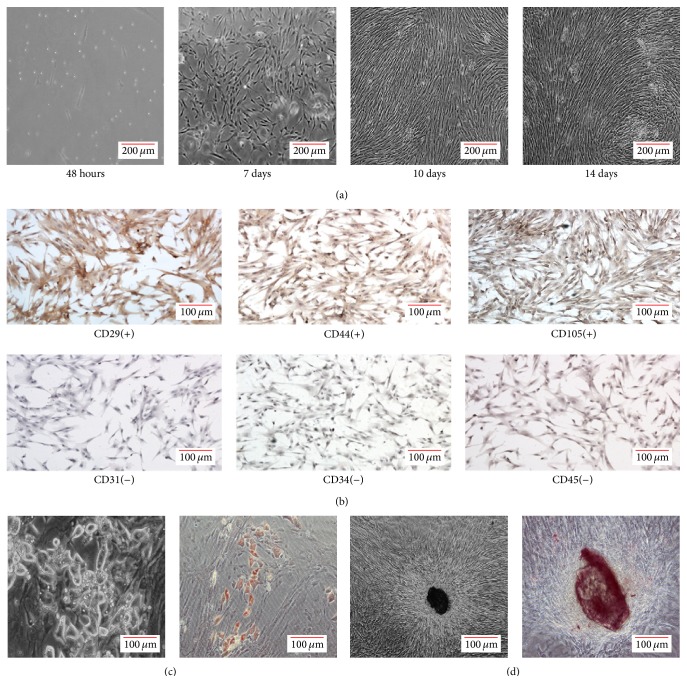
Isolation, culture, and identification of hUCMSCs. (a) Primary mesenchymal stem cells isolated from human Wharton's jelly formed colonies after 7 days, reached confluency after 10 days, and were in a vortex-like arrangement after 14 days. (b) The adherent cells were stained positively for mesenchymal markers CD29, CD44, and CD105 but were negative for hematopoietic lineage markers CD34 and CD45 and endothelial cell marker CD31. (c) After adipogenic induction for 2 weeks, vacuole-like changes were observed under a microscope (left, ×40), and the cells were stained by oil red O (right). (d) After osteogenic induction, refractile substances were observed in cell colonies (left, ×40), and small round nodules were detected by Alizarin red S staining (right).

hUCMSCs isolated from Wharton's jelly were maintained under standard culture conditions, and the primary and passaged cells both exhibited adherence to plastic (Figure 1(a)). Immunohistochemistry showed that the adherent cells were stained positively for mesenchymal markers CD29, CD44, and CD105 but were negative for hematopoietic lineage markers CD34 and CD45 and endothelial cell markers CD31 (Figure 1(b)). After adipogenic induction for 2 weeks, lipid droplets were found in the cytoplasm, indicating that the cells had differentiated into the fat cells (Figure 1(c)). In addition, during the osteogenic induction, there was little change in cell morphology, but refractile substances were observed in the cell colonies. Calcium accumulation was found after being induced for 2 weeks as small round Alizarin red-positive nodules in the cells (Figure 1(d)). Thus, the hUCMSCs demonstrated multipotency.

### 3.2. hUCMSCs Have an Odontogenic Differentiation Potential

**Figure 2:**
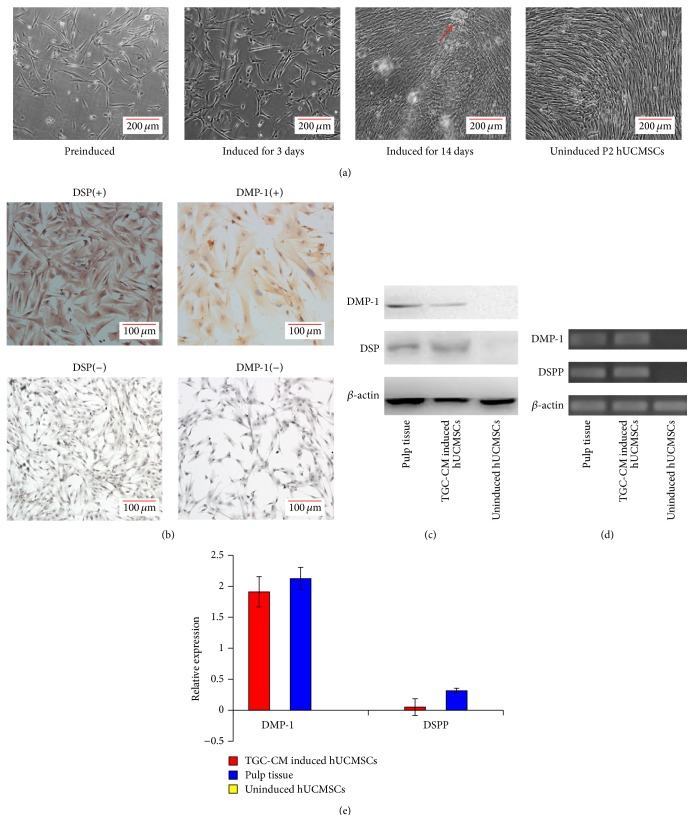
TGC-CM induces hUCMSCs into odontoblast-like cells* in vitro*. (a) After hUCMSCs were induced in TGC-CM for 3 days, there was little change in the cell morphology. After 14 days, the TGC-CM-induced hUCMSCs grew well and calcified areas were observed (arrow), but the morphology was similar to uninduced cells. (b) Odontoblast marker proteins DSP and DMP-1 were detected in TGC-CM-induced hUCMSCs by immunocytochemistry, but not in uninduced hUCMSCs. (c) Western blotting showed that TGC-CM-induced hUCMSCs and pulp tissue expressed DMP-1 and DSP, whereas uninduced hUCMSCs did not express these proteins. (d) and (e) Relative mRNA levels of DMP-1 and DSPP were determined by quantitative PCR.

After the hUCMSCs had been cultured in TGC-CM for 14 days, the cells grew well and exhibited a long fusiform shape with abundant cytoplasm, but the morphology did not change significantly during the induction procedure (Figure 2(a)). We also found that TGC-CM-induced hUCMSCs expressed both DSP and DMP-1 detected by immunocytochemistry and western blotting. These proteins were not expressed in uninduced hUCMSCs but were found in pulp tissue, indicating that the TGC-CM-induced hUCMSCs differentiated into odontoblast-like cells (Figures 2(b) and 2(c)). Next, quantitative PCR was used to compare the gene expression of* DSSP* and* DMP-1* in TGC-CM induced hUCMSCs and uninduced hUCMSCs. We found that* DSSP* and* DMP-1* gene expression was upregulated significantly in both TGC-CM-induced hUCMSCs and pulp tissue, whereas no expression was found in uninduced hUCMSCs (Figures 2(d) and 2(e)).

### 3.3. hTDM Provides an Odontogenic Microenvironment for hUCMSCs

**Figure 3:**
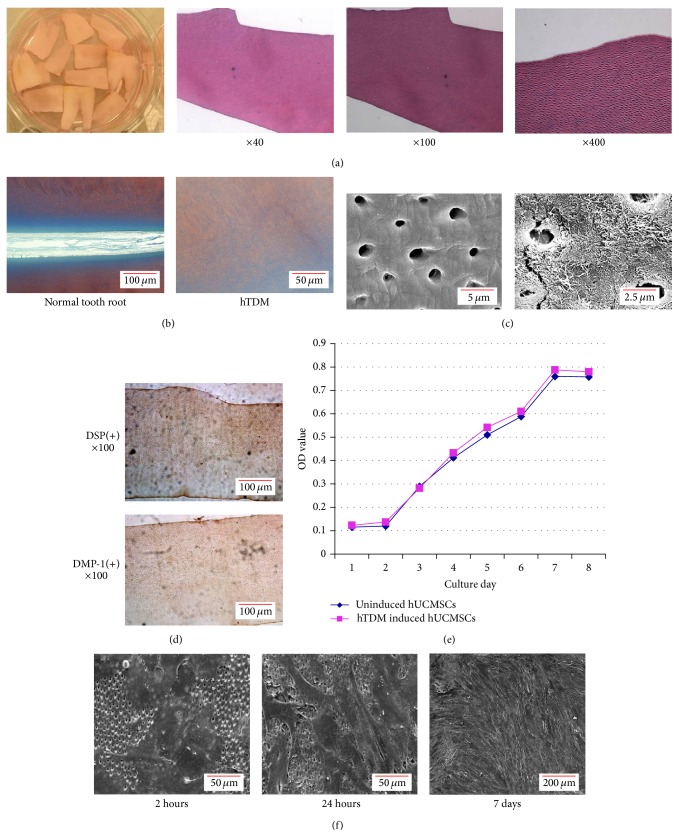
Preparation and identification of hTDM. (a) The gross appearance as well as HE staining of hTDM was observed under a microscope at various magnifications (×40, ×100, and ×400). HE staining showed the loose fiber tracts on the surface of the prepared hTDM. (b) Masson's tricolor staining showed dark red with gradual darker blue from distal to proximal pulp cavity dentin, where collagen fibers existed from low to high abundance in hTDM (right), just like staining in normal tooth root (left). (c) Scanning electron microscopic observation of the prepared hTDM. (d) Detection of DSP and DMP-1 in hTDM by immunohistochemistry. (e) Growth curves of hTDM-induced hUCMSCs and normal cultured hUCMSCs without hTDM as determined by MTT assays. (f) Observation of hUCMSCs after induction with hTDM for 2 hours, 24 hours, and 7 days under a scanning electron microscope.

The sectioned hTDM was stained with HE and Masson's trichrome. HE staining revealed loose fiber tracts on the surface of the prepared hTDM (Figure 3(a)). Masson's trichrome staining of the prepared hTDM was dark red with gradual darker blue from distal to proximal pulp cavity dentin, where collagen fibers existed from low to high abundance (Figure 3(b)). SEM observation further confirmed that the dentin tubules were fully exposed and the loose peritubular and intertubular fibers provided the space where hUCMSCs could keep contact with proteins and factors involved in dentin formation and thus provided an odontogenic microenvironment for the hUCMSCs (Figure 3(c)). Next, immunohistochemistry was used to determine whether DSP and DMP-1 were expressed in hTDM. As expected, hTDM was positive for DSP and DMP-1, especially around the dentin tubules, indicating that the dentin expressed DSP and DMP-1 (Figure 3(d)).

The growth curve detected by MTT assays of hTDM-induced hUCMSCs and normal cultured hUCMSCs without hTDM were similar. Both showed a latent phase for 24 to 36 hours, a logarithmic phase for 5 or 6 days, and a plateau phase after 7 days of culture (Figure 3(e)). And the SEM showed the hUCMSCs had adhered to the hTDM surface after induction for 2 hours, and then the cells began to spread after 24 hours and the whole hTDM surface was covered by cells after 7 days. These observations demonstrated that the hTDM had no effect on proliferation of the hUCMSCs (Figure 3(f)).

**Figure 4:**
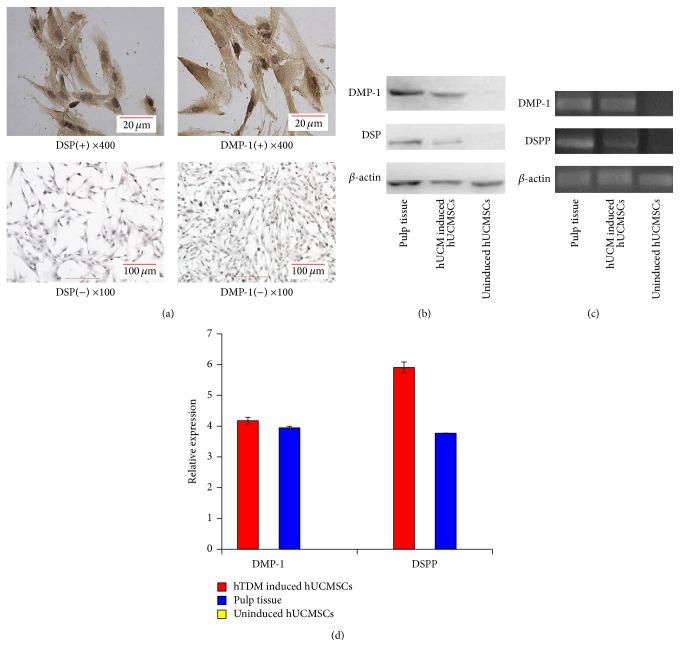
hTDM induces hUCMSCs into odontoblast-like cells* in vitro*. (a) Detection of DSP and DMP-1 in hTDM-induced hUCMSCs and normal cultured hUCMSCs without hTDM by immunocytochemistry. (b) Western blotting showed that hTDM-induced hUCMSCs and pulp tissue expressed DSP and DMP-1, while uninduced hUCMSCs did not express these proteins. (c) and (d) Relative mRNA levels of DMP-1 and DSPP were determined by quantitative PCR.

Then immunocytochemistry and western blotting proved DSP and DMP-1 exist in hTDM-induced hUCMSCs (Figures 4(a) and 4(b)). We also found that hTDM-induced hUCMSCs expressed* DSPP* and* DMP-1*, whereas the uninduced hUCMSCs did not express these genes. Despite the low level of* DSPP* expression, the differences in* DSPP* and* DMP-1* expression between hTDM-induced hUCMSCs and uninduced hUCMSCs were statistically significant (Figures 4(c) and 4(d)).

### 3.4. Dentin Regeneration by Subcutaneously Implanting hTDM-hUCMSC Composites

**Figure 5:**
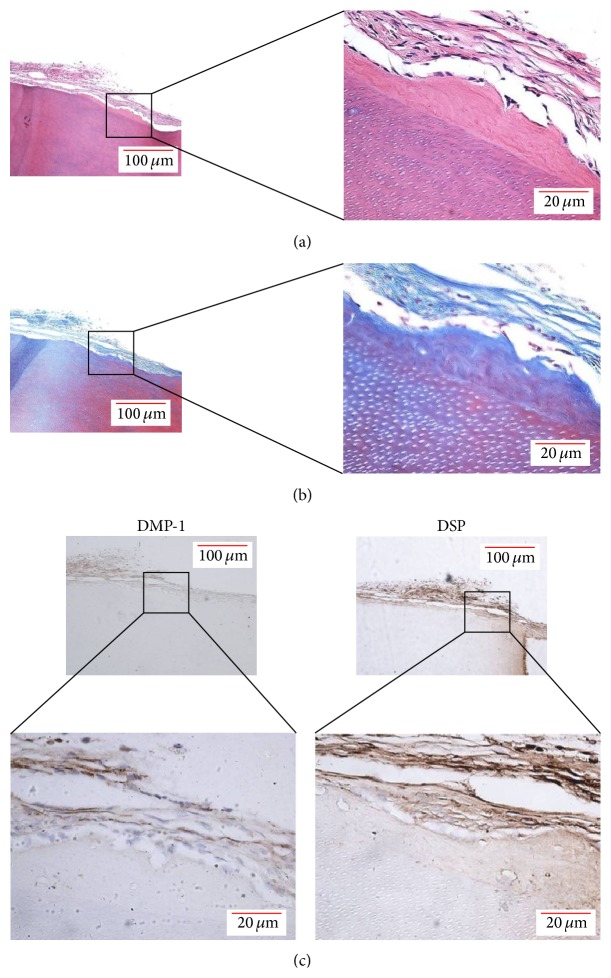
hTDM induces hUCMSCs into odontoblast-like cells* in vivo*. (a) HE staining of hTDM-hUCMSC composites after subcutaneous implantation into nude mice for 8 weeks. (b) Masson's tricolor staining of hTDM-hUCMSC composites after subcutaneous implantation into nude mice for 8 weeks. (c) Newly formed calcification and adhesive cells on hTDM were positive for DSP and DMP-1 as detected by immunohistochemistry.

To investigate whether hUCMSCs are suitable seed cells for tooth regeneration, we implanted hTDM-hUCMSC composites* in vivo*. The hTDM-hUCMSC composites were harvested after subcutaneous implantation into 20 nude mice for 8 weeks. We found no swelling or inflammation in the tissues around the implants. Furthermore, the implants maintained their original appearance without any degradation. HE staining showed newly formed calcification on the hTDM with multiple layers of cells but no inflammatory cells (Figure 5(a)). Masson's trichrome staining also showed newly formed calcification (Figure 5(b)). Importantly, the newly formed calcification and adherent cells were positive for DSP and DMP-1 as detected by immunohistochemistry (Figure 5(c)). These results showed that hUCMSCs can be induced into odontoblast-like cells by hTDM* in vivo*.

## 4. Discussion

The hUCMSCs can be isolated from various regions of the umbilical cord. These regions include Wharton's jelly, umbilical vein subendothelium, and the perivascular region [32]. Wharton's jelly is a primitive mucous connective tissue that surrounds the umbilical cord arteries and vein. The mesenchymal stem cells from Wharton's jelly have been confirmed as a primitive stem cell population [33]. In addition, stem cells from Wharton's jelly have a better multiple differentiation potential than those from other regions of umbilical cord [34, 35]. Therefore, we isolated MSCs from Wharton's jelly of the human umbilical cord and applied them for tooth regeneration.

The niche plays an important role in stem cell proliferation and differentiation. Specific niches participate in regulating the asymmetric divisions of stem cells [36, 37]. Thus, to induce hUCMSCs into odontoblast-like cells* in vitro*, it is important to provide a microenvironment that mimics the specific niche of tooth morphogenesis and facilitates odontogenic differentiation. Previous studies have demonstrated that TGC-CM provides a microenvironment enriched with regulating factors for tooth morphogenesis, which enhanced odontogenic differentiation of dental as well as nondental stem cells [38, 39]. Tooth morphogenesis is regulated by sequential and reciprocal interactions between the epithelial and mesenchymal tissues. Moreover, the signals from the oral epithelium play a decisive role in tooth morphogenesis, which act on ectomesenchyme cells to initiate and maintain tooth morphogenesis [40]. When developing tooth germ cells are cultured in the culture medium, the interactions between the epithelial and mesenchymal cells result in secretion of various factors including Wnt, fibroblast growth factor, transforming growth factor-*β*, and bone morphogenetic proteins [41, 42]. After inducing hUCMSCs with TGC-CM, these factors initiate and maintain odontogenic differentiation.

Tooth regeneration mainly focuses on three aspects: seed cells, scaffolds, and growth factors [43]. Seed cells for tooth regeneration should be able to differentiate into tooth-specific cells and form dentin, enamel, cementum, and alveolar bone in a suitable scaffold [44]. To determine whether hUCMSCs are suitable for tooth regeneration, we carried out* in vitro* and* in vivo* experiments using cocultured hUCMSCs with scaffolds. There are various scaffolds for tooth regeneration such as collagen, polyglycolic acid, and polylactic acid [45]. Among them, TDM is a newly developed scaffold for tooth regeneration. Prepared hTDM maintains the major structure of dentin tubules, which is essential for dentin regeneration. In addition, hTDM expressed DSP and DMP-1 which have been demonstrated to play critical roles in dentinogenesis [46]. Thus, hTDM not only serves as a scaffold, but also provides an odontoblastic microenvironment for stem cells [30, 47]. Therefore, hTDM is regarded as an available scaffold for tooth regeneration. Our results showed that hUCMSCs can be differentiated into odontoblast-like cells by hTDM* in vitro*, and that the proliferation rate of hUCMSCs was not altered after combining with hTDM. Furthermore, newly formed calcifications were observed after hTDM-hUCMSC composites were implanted subcutaneously into nude mice for 8 weeks. Moreover, the newly formed calcifications were positive for DSP and DMP-1 as detected by immunohistochemistry, revealing that the newly formed calcifications were likely to be dentin-like matrix. And unsurprisingly, the hUCMSCs without hTDM in control groups could not differentiation into odontoblast-like cells whether* in vitro* or* in vivo*. Therefore, we concluded that hUCMSC can be induced into odontoblast-like cells that secrete a dentin-like matrix* in vivo*, suggesting that hUCMSCs are a suitable and practical cell type which could be applied in tooth regeneration.

DSP (encoded by gene* DSPP*) is related to differentiation and mineralization of odontoblasts [48]. In addition, DMP-1 (encoded by the gene* DMP-1*) controls nucleation of calcium phosphate polymorphs and promotes pulp stem cell differentiation into odontoblasts [49]. DSP and DMP-1 are generally regarded as odontoblast-specific markers to identify induction of odontoblast-like cells [50, 51]. In the present study, hUCMSCs induced by TGC-CM or hTDM* in vitro* or* in vivo* were found to express DSP and DMP-1 with upregulation of* DSPP* and* DMP-1* gene expression to levels similar to those in pulp tissue.

Although we have confirmed the hUCMSCs have an odontogenic differentiation potential, there are some limitations and challenges should be concerned. Although the sources of hUCMCSs are rich, the differentiation efficiency should be improved. And how to maintain cell stability and consistency should also be considered. For tooth regeneration research, further studies like distinguishing odontogenic differentiation from osteogenic differentiation are needed. And regenerating the whole tooth with hUCMSCs including periodontal ligament, dental pulp enamel, cementum, and dentin should also be studied in the near future.

## 5. Conclusion

In this study, we show that the hUCMSCs have an odontogenic differentiation potential to differentiate into odontoblast-like cells in an odontogenic microenvironment provided by TGC-CM and hTDM* in vitro*. Furthermore, hUCMSCs deposited a dentin-like matrix when combined with hTDM* in vivo*. Overall, hUCMSCs may be a new therapeutic cell source for tooth regeneration.

## Figures and Tables

**Table 1:** Forward (F) and reverse (R) primer sequences for target and reference genes.

Gene name	Primer sequence (5′-3′)	Fragment length (bp)	Accession number
*β*-actin	F: GAAGATCAAGATCATTGCTCCT	111	NM_031144.2
	R: TACTCCTGCTTGCTGATCCA		
DMP-1	F: AAGATCAGCATCCTGCTCAT	91	NM_004407.3
	R: CTTCAGAATCCTCAGATTCAT		
DSPP	F: GAATAGAGGACACCCAGAAG	165	NM_014208.3
	R: CTTTCCCAACTTCTTTGGTAAT		
